# Flexible updating of dynamic knowledge structures

**DOI:** 10.1038/s41598-019-39468-9

**Published:** 2019-02-19

**Authors:** Franziska R. Richter, Paul M. Bays, Priyanga Jeyarathnarajah, Jon S. Simons

**Affiliations:** 1 Cognitive Psychology Unit, Institute of Psychology, Leiden University, Leiden, The Netherlands; 2 Department of Psychology, University of Cambridge, Cambridge, United Kingdom

## Abstract

Schemas are knowledge structures that allow us to make efficient judgments about the world without the cost of memorizing every detail of previous experiences. It has long been known that schemas can enhance long-term memory for related information. The usefulness of schemas, however, critically depends on their adaptability: how flexibly a schema can be updated according to changing environmental conditions. Prior consolidation of a schema supports new learning of schema-consistent information. Yet, the effect of consolidation on inconsistent information, and how schemas may be subsequently updated, are not well understood. It is difficult to track the dynamic updating of knowledge structures with traditional memory measures. Here, using a continuous-report paradigm, we were able to show that schematization increases incrementally with consolidation and that the strength with which schemas are initially established predicts schema-guided responding in a later test. Critically, schema updating in response to inconsistent information was more pronounced in a group which was given time to consolidate compared to a group that was not given time to consolidate. Importantly, the later group reverted back to the no longer relevant schema, indicating that systematic bias towards old information, rather than increased forgetting, underlies reduced memory for schema-inconsistent information.

## Introduction

The term ‘schema’ refers to complex knowledge structures within memory that connect overlapping elements of information. Schemas are believed to support the retention of information derived from a multitude of individual episodic memories, and to help us generalize from previous experiences and predict the outcomes of future events^1,2^. It has long been known that memory schemas are beneficial for the remembering of schema congruent versus incongruent information^3–5^. This effect has been attributed to both enhanced deep encoding^6^ and increases in schema-consistent guesses^7,8^. During encoding, schema congruency can accelerate the onset of memory formation processes^9^. During retrieval, the automatic activation of related information in response to a retrieval cue enhances retrieval success^1^. Thus, schemas contribute to memory performance by influencing processing during both encoding and retrieval.

The development of knowledge structures such as schemas relies heavily on consolidation, the process by which memories are not only stabilized but also transformed (i.e., abstracted or generalized) over time^10,11^. Consolidated schemas provide us with a framework to predict outcomes according to our knowledge of events that fit the existing schema. Models of reinforcement learning regard the drive to optimally predict outcomes of the world as one of the main sources of learning^12^. Erroneous predictions due to inconsistent information (i.e., unexpected outcomes) may be one source of such learning^13^. Similar mechanisms have been postulated in the long-term memory literature: predictive coding models of memory are based on the idea that learning results from divergences between experience-based predictions and observed outcomes^14,15^.

Learning inconsistent information provides a unique challenge because our cognitive system has to recognize that this information is not simply the result of noisy consistent data, but instead represents a shift in the environment. Understanding the fate of inconsistent information is, for example, critical in the context of education, where a student’s prior incongruent beliefs might hinder new learning^16^. Because it is necessary to notice the inconsistency between the pre-existing schema and current incoming information, efficient learning of inconsistent information should also require a stable pre-existing schema. Therefore, the process of *schema-updating* (adapting a schema in the face of new information) should heavily rely on consolidation, as only with consolidation the communalities between events will have been extracted, allowing for the detection of discrepancies. However, the effect of consolidation-related schema stabilisation on the successful learning of novel inconsistent information is not well understood. Findings that suggest a role of an existing stable memory representation in the learning of inconsistent information have recently been reported by Richards and colleagues^17^, who used a water-maze task in which mice had to learn to swim to platforms that clustered according to a location schema. The authors found that a 30- vs. 1-day consolidation period augmented the formation of schemas: mice were better at finding schema-consistent platforms with the longer compared to the shorter delay after learning a location schema. Furthermore, consolidated schema memories increased sensitivity to information that was inconsistent with the schema: mice initially displayed higher errors during these trials, but more learning of this information was evident in a later final test. That is, the authors found stronger updating during the learning of inconsistent information when learning occurred in the context of a consolidated versus an unconsolidated schema. While the study by Richards *et al*.^17^ thus indicates that (at least in an animal model) consolidation is of critical importance for schema updating, the fate of inconsistent information in the unconsolidated condition remains unclear. Is unconsolidated inconsistent information forgotten at a higher rate, because it does not fit the schema, or does it not become accommodated into the new schema at the same rate, due to systematic bias? In other words: Is the new schema not updated as successfully if the prior schema was not consolidated?

Understanding how schemas and their consolidation affect memory requires a detailed characterization of how individual episodes are transformed into conceptual representations, and how existing schemas are modified to accommodate new information. Ghosh & Gilboa^2^ identified *adaptability* as one of the defining features of schemas, which refers to the malleability of schemas to incorporate additional information, either by inclusion of new information to the existing schema structure, or by modifying the existing structure if necessary. Yet, the process of schema adaptation manifests incrementally, with gradual small changes to existing schemas. Such gradual dynamic updating is difficult to capture with conventional categorical memory measures^2,18,19^ and has therefore so far largely escaped study. The recent development of continuous long-term memory measures^20–23^ now allows assessment of small changes in the objective quality of memories, a critical requirement to study the construction and updating of schemas. The current study thus aimed to track the development and modification of schemas across a several day period. Using continuous memory measures to capture small changes in memories over the course of schematization, we can establish the dynamics of schema updating in the face of inconsistent newly learned information and its dependency on consolidation.

Specifically, in the current study we used spatially distributed locations to track the development and manipulation of memory schemas in humans. Participants learned two original location schemas for different categories of objects during an initial training period. Subsequently, one of the locations schemas was changed, unbeknownst to the participants, such that newly learned information was inconsistent with the original schema. We assessed how consolidation of the initially learned schema affected this processing of schema-consistent and inconsistent information over experimental sessions, by comparing a group that was presented with this information before, and another group that was presented with this information after a consolidation period. Importantly, using continuous measures of memory, we were able to test the effect of consolidation on the development and updating of schematic memories. Moreover, this approach also allowed us to answer the critical question of what happens to inconsistent information that is learned prior to, or after a schema has been consolidated. The following hypotheses were assessed: first, we predicted that progressive schema development would be observed over the course of the three experimental sessions, with the strongest evidence of schematization on the last day of testing. Second, we tested the central hypothesis that the introduction of schema-inconsistent information would lead to enhanced schema updating if this information is introduced *after* vs. *before* consolidation. Lastly, we assessed how the strength with which a schema is initially established influences later memory for schemas that remain consistent, versus schemas that become inconsistent depending on consolidation.

## Results

### Analysis approach

**Figure 1:**
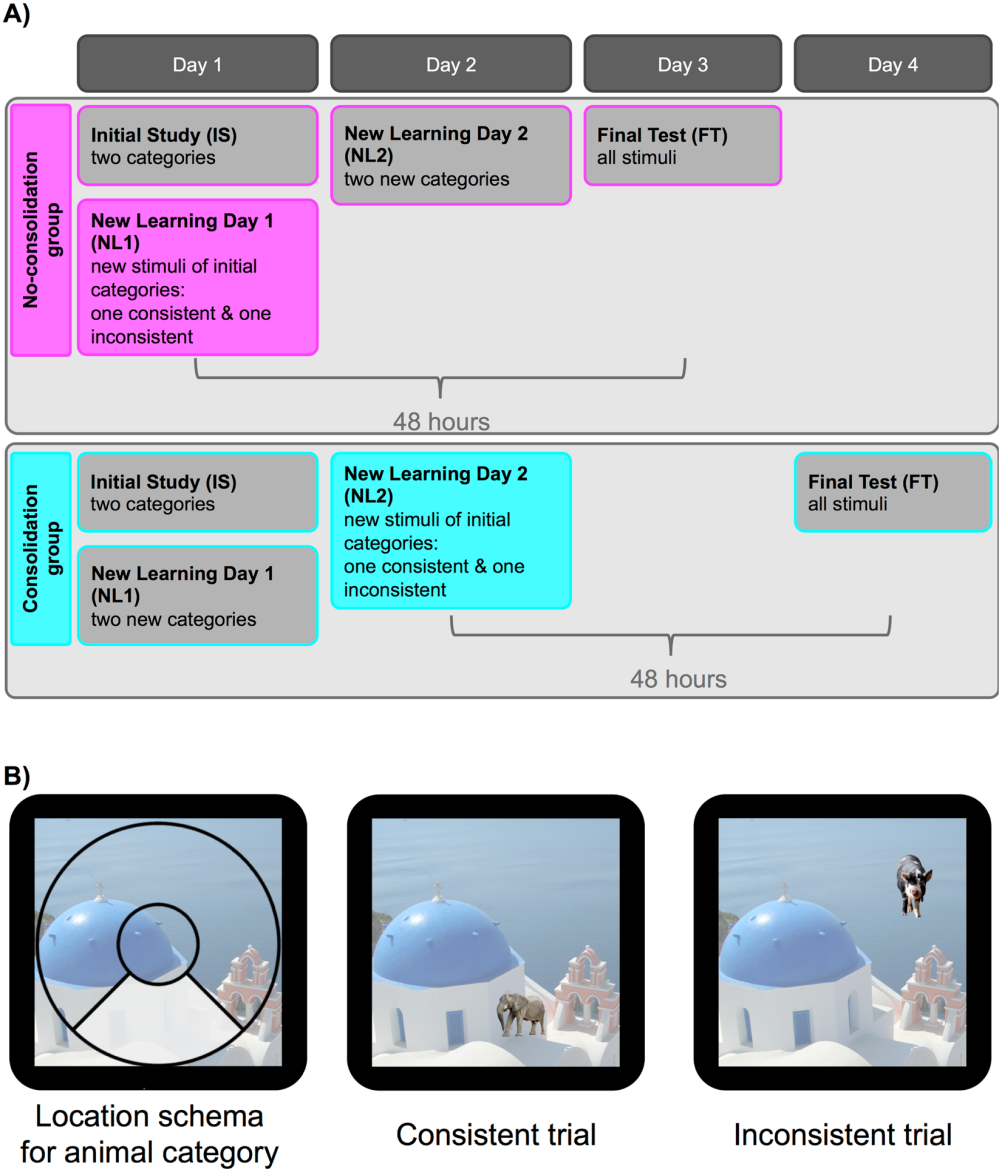
(**A**) Experimental phases for both groups across the sessions of the experiment. The no-consolidation group learned two categories of stimuli in the IS phase on day 1 and learned new stimuli of the same two categories during NL1. This group learned two new categories (the two categories that were not learned on day 1, or ‘irrelevant’ categories) on day 2, and came back for the final memory test on day 3. The consolidation group also learned two categories during the IS period on day 1, but in contrast to the no-consolidation group subsequently learned two new categories (the two categories that were not learned in the IS phase, or ‘irrelevant’ categories). They were presented with new stimuli of the same two categories that were learned during IS only on day 2, and came back for a final memory test on day 4. In NL1 for the no-consolidation group and NL2 for the consolidation group one of the two categories learned changed their schema mean: the mean shifted by 90 degrees clockwise compared to what it was in the IS phase. This category is referred to as the inconsistent category. (B) Left: Circle segment associated with the animal schema. Most of the animals are presented within the shaded area (not shown during the experiment). Middle: Inside-quadrant animal trial; the stimulus is presented within the area shown on the left. Right: Outside-quadrant animal trial; the stimulus is not presented within the area specified on the left. Image of Oia Church is a cropped version of the image from wikipedia.org, licensed under the Creative Commons Attribution-ShareAlike 3.0 License (https://creativecommons.org/licenses/by-sa/3.0/). All other images (pig and elephant, backgrounds removed) were retrieved from pixabay.com, licensed under a Creative Commons CC0 License (https://creativecommons.org/publicdomain/zero/1.0/deed.en).

The detailed procedures are described in the Methods section, but to provide an overview of the analysis approach, Fig. 1 gives an illustration of the design and Table 1 lists the abbreviations used in this study. Participants were presented with stimuli from different categories in several sessions across 3–4 days. On the first two days participants completed a total of 12 blocks with 28 stimuli each. Each block contained a study phase and a test phase. In the study phase participants were presented with each trial-unique stimulus once for 4 s and were instructed to remember its location on an invisible circle (no overt response was required from participants in the study phase). In the test phase participants had 6 s to recreate the learned location on the circle using continuous response options. For each of four categories stimuli were clustered within a category specific quadrant along the invisible circle (location schemas). For each category most of the stimuli were presented within this category-specific circle location (inside-quadrant trials, 22 out of 28 items per block). However, a subset of trials did not follow this category-specific location schema (outside-quadrant trials, 6 out of 28 items per block). Moreover, across the different testing sessions, the categories themselves were either consistent (the 90 degree segment associated with this category remained constant across sessions), or inconsistent (between sessions the 90 degree segment associated with this category shifted once by 90 degrees clockwise). Half of the participants followed the protocol for the no-consolidation group, meaning they experienced this shift in the category schema on day 1, before consolidation. The other participants made up the consolidation group, and only learned this inconsistent information on day 2, after 24 hours time for consolidation. All stimuli were tested a second time in the final session on the last day of testing.

**Table 1:** Glossary.

IS	Initial Study phase on day 1.
NL1, NL2	New learning phase on day 1 (following IS) or day 2, respectively.
FT	Final Test during the last session.
Schema mean	The average location of stimuli across all trials for a category.
Consistent Categories	The same schema mean was used for these categories throughout the experiment.
Inconsistent Category	Category for which the schema mean shifted by 90 degrees in the NL phase (NL1 or NL2, depending on group assignment).
New/Irrelevant Categories	The two categories learned during NL1 or NL2 that are distinct from the categories learned during IS.
Inside-quadrant trials	Within the current category, the location of this item falls within ±45 degrees of the schema mean.
Outside-quadrant trials	Within the current category, the location of this item does *not* fall within ±45 degrees of the schema mean, but is at least 30 degrees removed from the schema-quadrant.
Relevant Categories/Stimuli	Items from the same categories as learned during IS (including all items learned during IS and NL phases).
Critical Items	Only those items from the relevant categories that were learned during New learning, exactly two days before the Final Test. These items were learned in NL1 for the no-consolidation group, and during NL2 for the consolidation group. For one of these categories the category mean remained consistent (consistent category), for the other one the category mean changed (inconsistent category).

### Schematization effects over the course of the experiment

#### Absolute Error

To test the hypothesis that schematisation occurs over time, and increases throughout the experiment, we used two approaches. In a first step we investigated whether outside- compared to inside-quadrant trials would be associated with lower response accuracy in each of the different phases of the experiment (Initial Study, New Learning, and Final Test phase). Trial-specific estimates of accuracy were obtained by measuring the absolute error, that is the absolute difference between the target location and the recreated location (ranging from 0–180 degrees, with higher values indicating less accurate responses). We hypothesised that over time, a difference in absolute error between outside- and inside-quadrant trials should be observed, and that this difference would be most pronounced in the Final Test. For this analysis we specifically focused on the consistent condition for which the *schema mean* (i.e., the average location across all trials for a given category) remained constant throughout the experiment. This analysis enabled us to compare performance across the different experimental phases, without introducing unnecessary noise due to the change in schema mean and consequently hypothesized schema updating that would be expected in the inconsistent category.

**Figure 2:**
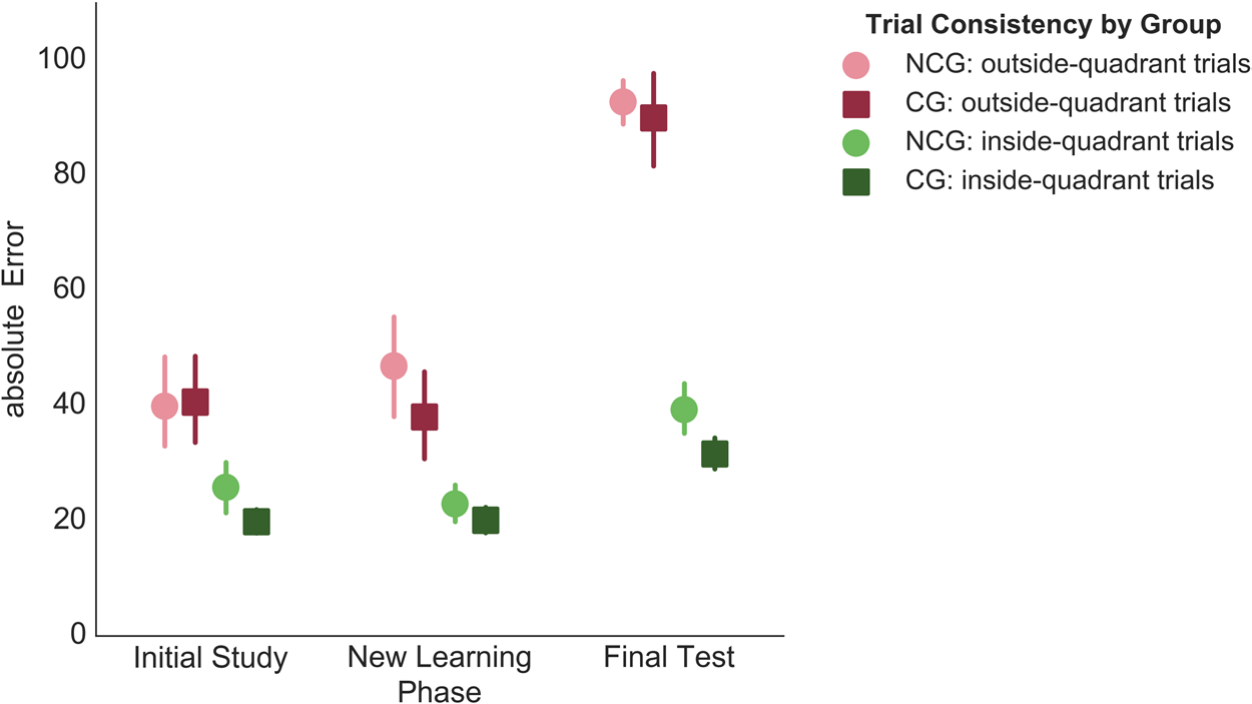
Mean absolute error in inside-quadrant (green) and outside-quadrant (red) trials across experimental phases in the consistent category condition, for the no-consolidation group (lighter shades, circles) and the consolidation group (darker shades, squares). Error bars reflect standard errors of the mean.

Given that the same computational model might not fit equally well in the different phases of the experiment (as errors differed substantially between the phases, especially day 1 and 2 phases and the Final Test), we focus on unmodelled data in the current analysis (see below for a model based analysis of the Final Test data). Performance in the outside- and inside-quadrant trials is plotted in Fig. 2 for the different phases of the experiment: Initial Study (IS), New Learning on day 1 or 2 (NL1 or NL2), and Final Test (FT). (In this figure ‘New Learning’ refers to NL1 for the no-consolidation group and NL2 for the consolidation group).

As can be seen visually, performance in the outside-quadrant trials dropped below that of the inside-quadrant trials early in the experiment. Moreover, this effect seemed to increase over time. A mixed ANOVA with factors phase (IS, NL [NL1 for no-consolidation group and NL2 for consolidation group] and FT), trial quadrant (inside vs. outside) and group (consolidation group versus no-consolidation group) was conducted. IS included only trials from the category that later remained consistent (56 trials). FT trials included in this analysis were responses to trials that were learned in the relevant NL phases (56 trials, ‘critical items’, see Table 1). That is, responses to the same stimuli were included for NL and FT, but once these responses were from the NL test phase, and once they were given 48 hours later, in the Final Test.

The ANOVA revealed significant main effects of phase, *F*(2,124) = 236.115, *p* < 0.001, *partial η*^2^ = 0.911, with numerically highest absolute error in the Final Test, and trial quadrant, *F*(1,62) = 382.518, *p* < 0.001, *partial η*^2^ = 0.861, indicating overall higher absolute error values in outside-quadrant trials. These main effects were further qualified by the predicted significant interaction between phase and trial quadrant, Sphericity assumption violated, *ε* = 0.879, *F*(2,124) = 106.357, *p* < 0.001, *partial η*^2^ = 0.632, indicating the largest difference between inside- and outside-quadrant trials in the Final Test. Moreover, there was a significant interaction between group, phase and trial quadrant, *F*(2,124) = 3.426, *p* = 0.036, *partial η*^2^ = 0.052.

Follow-up analyses revealed that while there were no significant interactions between group and trial quadrant for phases IS and NL (all *ps* ≥ 0.160), there was a marginally significant interaction for the FT data, *F*(1,62) = 3.399, *p* = 0.070, *partial η*^2^ = 0.052. This effect was due to a marginally larger difference between inside- and outside-quadrant trials in the consolidation group, than the no-consolidation group, *t(6*2*)* = 1.844, *p* = 0.070, equal variances assumed. Nevertheless, inside-quadrant trials displayed lower absolute error than outside-quadrant trials for both groups in the FT, non-consolidation group: *t*(31) = 23.042, *p* < 0.001, consolidation group: *t*(31) = 21.571, *p* < 0.001, consistent with predictions. Moreover, when considering schematization across phases, the trial-quadrant effect was increased in the FT compared to performance on the same items in the NL phase (*t*(63) = 11.362, *p* < 0.001, and compared to the IS phase (*t*(63) = 16.404, *p* < 0.001. However, there was no significant difference in this effect between IS and NL, *t*(63) = 1.093, *p* = 0.279. Thus, the results from the analysis of absolute error during inside versus outside-quadrant trials indicate that participants learned the location schemas over the course of the experiment, that they performed better in trials that were within the category quadrant than trials that were outside and that this difference was most pronounced after a 48 hour delay, compared to testing immediately after the study blocks in IS and NL.

#### Modelling

We also used a second approach to test the hypothesis that participants developed a schema in the current experiment. In this second step we used computational modelling to test whether a model that explicitly includes the category specific location schema describes the performance during the Final Test better than a model that does not take the schema into account. This way, we tested the hypothesis that participants would be biased towards the schema mean^24^. We therefore compared a ‘standard’ mixture model comprising a von Mises distribution centred around the target values and a uniform component^23^ with a Model that also included a von Mises distribution centred around the category specific schema mean (‘standard + schema mean’ model). This second von Mises distribution was included to capture responses that were more consistent with the schema mean than the trial-specific target value. (The approach is similar to that of modelling non-target responses in previous work^25^). Modelling was performed on data pooled across participants to enhance stability of the model, and modelling was performed separately for both groups. As with the absolute error analysis described above, we again focussed only on the consistent condition. Model fit was assessed via Akaike information criterion (AIC) and Bayesian information criterion (BIC). In line with our hypothesis that participants established a schema, modelling the errors with the ‘standard’ mixture model (Model 1) of a von Mises (circular normal) distribution and a uniform distribution fit the data less well than a model that also included an additional von Mises distribution centred around the schema mean (‘standard + schema mean’ model, Model 2) in both no-consolidation group (AIC difference: 1027; BIC difference: 1022) and consolidation group participants (AIC difference: 1264; BIC difference: 1259). Thus, both the analysis of performance in terms of absolute error in outside- versus inside-quadrant trials, as well as the result of the model comparison, revealed that participants show evidence of schematization.

### Increased schema updating with consolidation

#### Modelling

**Figure 3:**
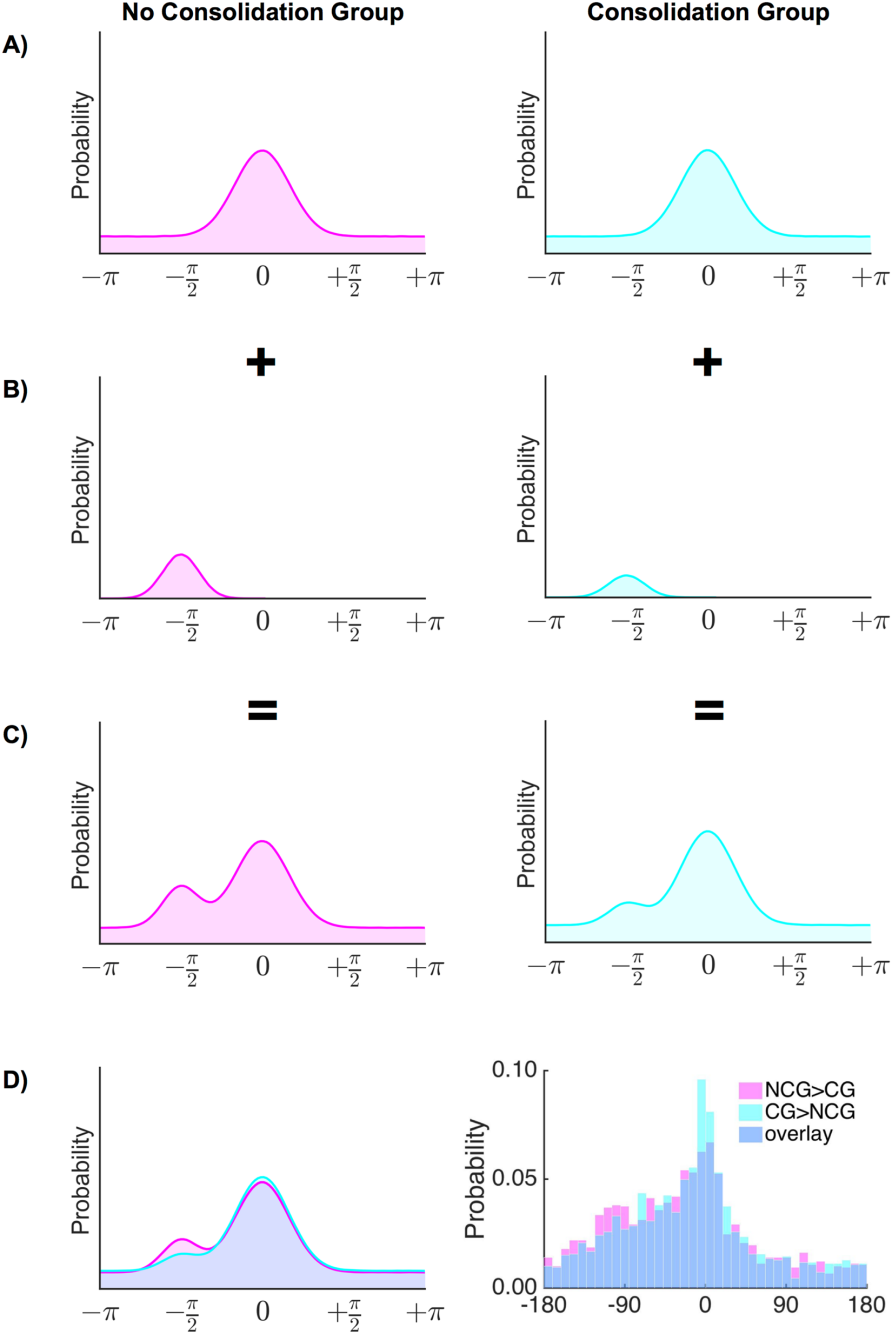
(**A–D**) Schematic description of the hypothesized model components used for both the no-consolidation group (magenta) and the consolidation group (cyan) in Model 4. (**A**) Standard mixture model. (Note that (**A**) by itself is equivalent to Model 3). (**B**) In Model 4 a number of trials are described by a von Mises distribution centred on the old schema mean (on average −90 degrees from the target value). It is predicted on some trials that participants will revert to the old schema mean instead of random guesses or responding with the target value. This effect is predicted to be stronger in the no-consolidation group. (**C**) Hypothesized resulting distribution for trials of the inconsistent category, as a combination of the standard mixture model (**A**) and an additional von Mises distribution centred on the old schema mean (**B**). (**D**) Predicted (left) and observed (right) error distribution in the inconsistent category for the consolidation group (cyan, CG) and the no-consolidation group (magenta, NCG) overlaid on each other (overlap plotted in blue). Note the increased number of responses around the −90 degree location for the no-consolidation compared to the consolidation group.

Having established that participants developed schemas during the experiment, we next assessed the central hypothesis that schema updating is enhanced with consolidation, and therefore a difference in ‘schema-updating’ should be detected between the consolidation group and the no-consolidation group. Specifically, our hypothesis states that the group that did not get a chance to consolidate before learning new schema-inconsistent information should have a less established schema, therefore a smaller prediction error in response to new inconsistent information and consequently, less updating of the memory schema. As a result, the no-consolidation group should show more evidence of being biased to the old, previously relevant schema mean in the FT. To test this hypothesis, we focussed on memory in the FT for ‘critical items’ of the inconsistent category, that is, the inconsistent category items learned during NL1 (no-consolidation group) and NL2 (consolidation group). (Or, in other words, items of the inconsistent category that were learned 48 hours before the FT). Before testing the hypothesis of whether more schema updating would occur in the consolidation group compared to the no-consolidation group, we first established whether our proposed model (a standard mixture model with an additional von Mises distribution centred on the old schema mean, see Fig. 3C; see Methods for detailed model specifications) indeed fit the data better than plausible alternative models, indicating that participants in fact use the old schema means on a subset of trials to guide their responses.

We compared 3 models: the ‘standard’ long-term memory mixture model (von Mises + uniform, Model 3; cf. Fig. 3A), a model including a von Mises distribution centred around the previously relevant/original schema mean in addition to the ‘standard’ model (‘standard + original schema mean’ model, Model 4, see also Fig. 3C), and a model which instead included a von Mises distribution centred on the updated/new schema mean (i.e., the schema mean that was relevant at the time of learning) in addition to the standard model (‘standard + new schema mean’ model, Model 5).

The results of the model fit analysis (Table 2) revealed that, consistent with predictions that participants sometimes revert to the old schema mean, Model 4 fit better than Model 3 or Model 5. Therefore, it seems that both in the consolidation group and in the no-consolidation group, the model was favoured which accounted for the possibility that participants sometimes used the ‘old’ (previously relevant) schema mean to guide their responses.

**Table 2:** Mean AIC and BIC differences between the preferred model (Model 4: ‘standard + original schema mean’) and the two alternative models (Model 3: von Mises + uniform, and Model 5: ‘standard + new schema mean’) in each group.

	Model 3 - Model 4		Model 5 - Model 4	
	AIC difference	BIC difference	AIC difference	BIC difference
no-consolidation group	572	566	537	537
consolidation group	505	500	478	478

#### Permutation test

In a next step we tested whether this reversion to the old schema mean would be more pronounced in the no-consolidation group than the consolidation group, a finding that would be indicative of less schema updating in the no-consolidation group (see Fig. 3D). Using an approach similar to that described by Schneegans and Bays^26^ we calculated for each trial the posterior probability that the response stemmed from each of the three mixture components [target response, old-schema mean (non-target) response, or guessing]. We again modelled the data across participants, and subsequently used permutation testing to assess the hypothesis that there would be fewer trials assigned to the old schema mean in the consolidation group (as this group would update their schema more strongly) compared to the no-consolidation group (which we predicted should show less schema updating). The results of Model 4 indeed indicated that responses in the no-consolidation group had a higher probability (*M* = 0.49) of being attributed to the old-schema mean than responses in the consolidation group (*M* = 0.32). This difference was significant as determined by a permutation test (*p* = 0.010, 5000 iterations, see Methods). Of note, the increased updating in the consolidation group was only observed in the Final Test, but not when memory was tested immediately after studying in the relevant NL phases. Here, the no-consolidation group (*M* = 0.03) had only a numerically slightly higher percentage of trials that were attributed to the old schema mean than the consolidation group (*M* = 0.00), and this effect was not statistically reliable when assessed via permutation testing (*p* = 0.563, 5000 iterations).

### Effects of initial schema strength on later performance

**Figure 4:**
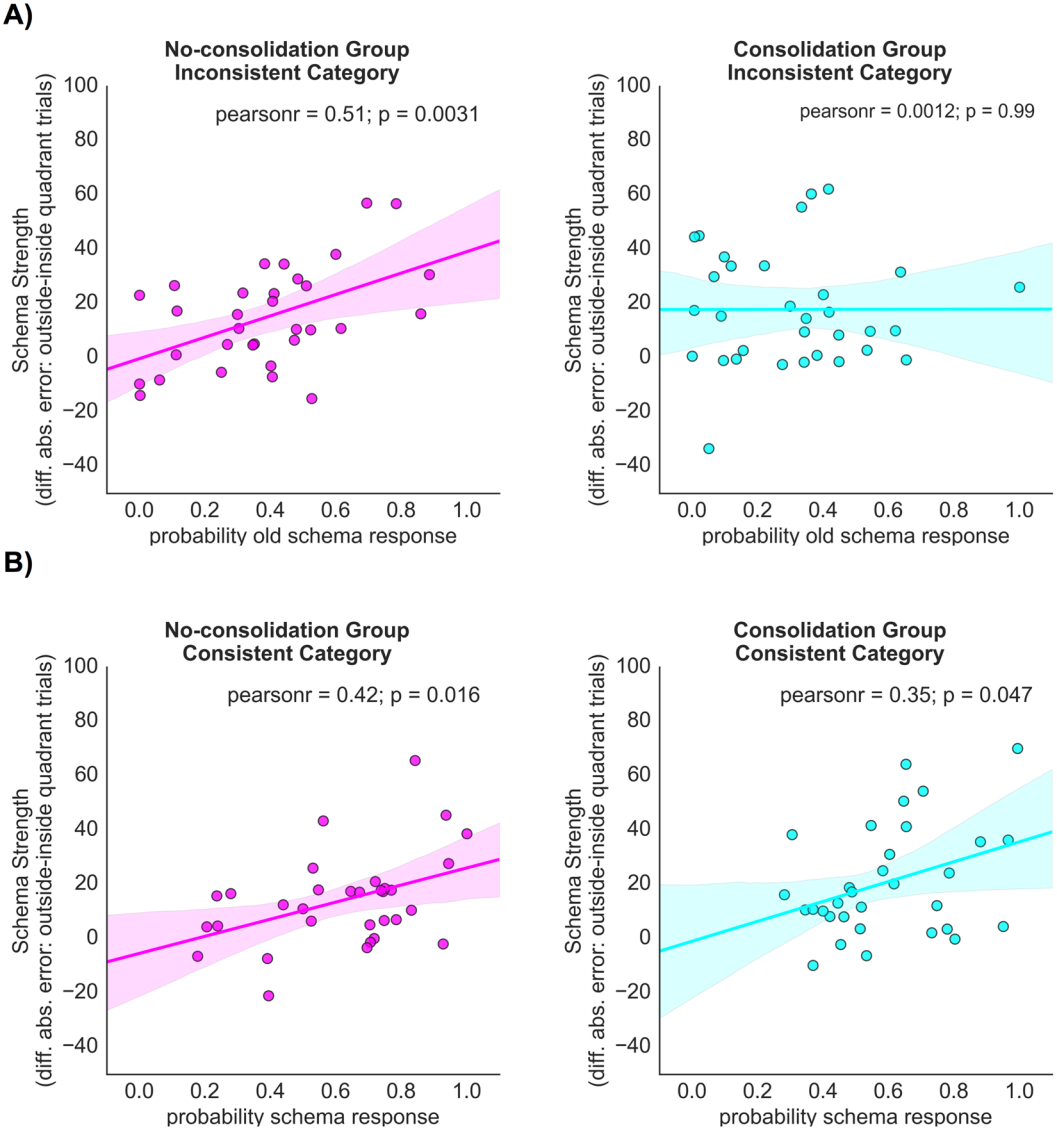
Relationship between initial schema strength (measured as the difference in absolute error between outside- and inside-quadrant trials in the Initial Study phase, for the consistent or inconsistent category, respectively) and the probability of responding consistent with the ‘old schema’ in the Final Test for trials of the inconsistent NL category (**A**) and the probability of responding consistent with the relevant schema in the Final Test for trials of the consistent category (**B**). Left: Correlations for the no-consolidation group. Right: Correlations for the consolidation group.

We next assessed whether the strength with which participants initially established the schemas would influence performance during the Final Test. For the purpose of this analysis we defined initial schema strength as the difference in absolute errors between outside- and inside-quadrant trials in the IS phase. (Analyses were conducted separately for the consistent and inconsistent category and schema strength was category specific, accordingly. Data was modelled on a single subject level to obtain subject specific values.) As a first prediction we hypothesised that a stronger initial schema should negatively affect performance in the inconsistent category for participants in the no-consolidation group. Specifically, we predicted that if a schema was strongly established during the Initial Study period, there should be a higher probability to revert to this schema during the Final Test, evident in responses that corresponded to the old schema mean (i.e., were assigned by the model to the old schema mean). The reasoning behind this prediction was that the combination of a strong initial schema combined with reduced schema updating in the inconsistent category in the no-consolidation group (due to lack of consolidation) would result in more ‘old schema mean responses’ during the Final Test. In contrast, in the consolidation group, which had consolidated the schema, a stronger initial schema should simultaneously lead to stronger prediction errors and thus stronger schema updating, which would counteract the tendency to revert to a strongly established initial schema. The probability of responding with the old schema mean was derived using the ‘standard + original schema mean’ model (Model 4) described above. Considering the relationship between initial schema strength and probability of responding with the old schema mean separately for both groups (see Fig. 4A), we found that, consistent with the above reasoning, the correlation between ‘initial schema strength’ and later likelihood for ‘old schema’ responses in the inconsistent category was significant for the no-consolidation group (*r* = 0.507, *p* = 0.003), but not for the consolidation group (*r* = 0.001, *p* = 0.995). Direct comparison of these two correlations revealed a significant difference (*z* = 2.12, *p* = 0.033). Thus, for the no-consolidation group there was a stronger relationship between the strength with which a schema was established during the IS period, and reverting to the old schema mean to guide responses to inconsistent category items in the Final Test, than for the consolidation group.

The enhanced tendency of ‘old schema’ responses in the FT for the no-consolidation group in the inconsistent condition could also be influenced by a numerically (though not significantly, see above) higher probability to already respond with the ‘old schema’ in the NL phase. To test whether bias towards the ‘old schema’ in the NL phase could (partly) explain the observed bias towards the ‘old schema’ in the FT we conducted a regression analysis, in which we added the probability of responding with the old schema in the relevant NL phase (NL1 for the no-consolidation group and NL2 for the consolidation group) in addition to considering initial schema strength. For the no-consolidation group the regression was significant, *F*(2,29) = 8.58, *p* = 0.001, *R*^2^ = 0.372. Here we found that, indeed, both initial schema strength (*β* = 0.457, *p* = 0.005), as well as the probability to respond with the ‘old schema’ in the NL phase (*β* = 0.343, *p* = 0.029) predicted ‘old schema’ responses in the FT. For the consolidation group, on the other hand no significant relationship was observed, *F*(2,29) < 1, *p* = 0.447, *R*^*2*^ = 0.054, similar to the results of the correlation analysis described above.

In a second step we also assessed performance in the consistent category. A strongly established schema should also affect performance here: specifically, a strong initial schema should lead to enhanced schema-like responding at Final Test. Since the schema does not require updating in the consistent category, we did not expect any differences between the two groups in the relationship between initial schema strength and schema-responses during the Final Test. The probability of schema-consistent responses was determined by employing the ‘standard + schema mean’ model (Model 2) described above. Consistent with predictions, there was a positive correlation between the strength of the schema during IS and the later probability of trials of the consistent category being attributed to the schema- distribution by the model (see Fig. 4B), for both the consolidation group (*r* = 0.354, *p* = 0.047), and the no-consolidation group (*r* = 0.422, *p* = 0.016). The difference between these correlations was not significant (z = 0.306, *p* = 0.759). Thus, for both groups there was a positive relationship between the strength with which the schema was established during the IS period, and schema-guided responses to consistent category items in the Final Test.

## Discussion

Schemas provide general knowledge structures that guide our everyday behaviour in many situations, and that also play a critical role in memory. The idea that consolidation is crucial for the establishment and modification of memory schemas is at the core of theories on knowledge acquisition. To be useful, schemas must be malleable and modifiable, a characteristic that has so far escaped direct observation due to the lack of resolution in previously available testing procedures. The research described here used a continuous report paradigm to track the development and modification of schema memories, providing novel insights into the nature of schematic knowledge acquisition.

The present data are consistent with the hypothesis that schema updating is dependent on the extraction of communalities between events with consolidation: schema updating in face of inconsistent information was enhanced when a relevant consolidated (in contrast to unconsolidated) schema existed. Moreover, our results indicate that while schematization was already detectable early in the acquisition phase, it was most pronounced after a delay, consistent with theoretical proposals on schema consolidation as well as recent findings in the animal literature which suggest that schemas develop over time^17^. We also demonstrate that more strongly established schemas are particularly resistant to updating when no consolidation is possible before new learning. When schemas remain consistent, however, this strongly established knowledge structure predicts schema-consistent responding later on, suggesting that in addition to abstraction processes with consolidation, the initial strength of the schema plays an important role in the way in which such knowledge influences later retrieval.

We interpret the finding that the no-consolidation group showed less schema updating than the consolidation group to indicate that consolidation strengthens schema-based predictions, and that violation of such predictions by the presentation of inconsistent information leads to enhanced ‘updating’ of the schema, or, in other words, more learning. Importantly, our first analysis found that both groups of participants successfully established schemas in the consistent category. Thus, the no-consolidation group (as well as the consolidation group) established schemas, but without an opportunity for schematization to progress during a consolidation period prior to learning conflicting information, they showed less updating of these schemas.

The finding that schemas appeared to guide responding as early as during the IS phase of day 1 may seem surprising, since at this point no (or very limited) consolidation had occurred. Moreover, previous research has suggested that inconsistent information might be recalled just as successfully as consistent information soon after encoding, and that both types of information are remembered better than neutral information only after 7 days, suggesting that schema consistency effects are noticeable only after a delay^27^. Our finding of a significant trial-quadrant (i.e., trial-consistency) effect early on could suggest that the continuous report paradigm used in the current work was potentially more sensitive to detect schema development than more traditional memory measures like the verbal recall task employed by Bonasia *et al*.^27^, who found these effects (worsened memory for inconsistent information) only after a delay.

An alternative explanation might be provided by recent findings by Antony, Ferreira, Norman, and Wimber^28^. The authors argue that retrieval can serve as a ‘fast route’ to memory consolidation. Given that participants retrieved memories during the test phase following each study phase in the current experiment, it is possible that memories were already somewhat consolidated/schematized after the Initial Study phase. Importantly, our finding for early schema effects do not conflict with the notion that consolidation on a longer time scale enhances schematization further. In fact, when comparing schematization for items in the FT that were learned 48 hours before they were tested, schematization was increased compared to performance on the same items in the NL phase. Thus, the added time seemed to benefit the establishment of schemas, and thereby enhanced the effect of schema consistency on memory performance.

Much evidence for the schematization of memories comes from findings that with schema consistency, correct recognition judgments, but also false alarms to similar items, increase^29,30^. We extend these findings by demonstrating that when participants exhibit decreased performance for schema-*inconsistent* information, they do not merely forget information, but substitute this information with a no-longer relevant schema, consistent with what has been argued to happen in Bartlett’s seminal folklore studies^3,27^. The present data demonstrate that the information that is substituted is consistent with the old schema, suggesting a lack of schema updating, rather than a general memory decay process, or a filling in of forgotten information by enhanced random guessing. This finding also has important implications for education, in case students possess prior incorrect or incongruent knowledge that conflicts with the to-be-learned information^16^.

We found that initial schema strength predicted schema-consistent responding in the Final Test for items of the consistent category regardless of group membership. This lack of a between-group effect is consistent with the idea that, if there is no need for schema updating (as was the case in the consistent category), schemas in both groups are consolidated (over the days before the Final Test), and initial schema strength is positively related with schema-based responding.

In contrast, the strength of the initial schema predicted a subsequent lack of schema updating in the inconsistent category only in the no-consolidation group. If the no-consolidation group was confronted with schema-inconsistent information, the lack of a (fully) developed schema meant that schema updating did not occur to the same degree as in the consolidation group. The old schema was instead recruited in the Final Test, particularly when this old schema was strongly established. For the consolidation group however, there was no relationship between initial schema strength and the degree of schema updating (even though this group showed a positive relationship between initial schema strength and schema-consistent responding for items from the consistent category). This finding suggests that consolidation after initial learning can protect participants from response bias towards an old schema due to its initial strength. Here, we assume that the stronger initial schema is compensated by a stronger ‘surprise’ signal (prediction error) when conflicting information is encountered, which then enhances the learning of the new schema, leading to schema updating. Why, then, was no *negative* (instead of a zero) correlation (indicative of enhanced updating) observed for the consolidation group in the inconsistent condition? We believe that because participants knew that half of the items included in the Final Test had been learned with the ‘original’ schema mean, in case they were unsure about an item they would equally often call upon the old and new schema to guide their responses, resulting in the observed null correlation.

With regards to the initial schema strength analysis, a reminder that, as stated in the methods section, there was a timing difference between IS and FT for the two groups: while IS took place on day 1 for both groups, the Final Test occurred on day 3 for the no-consolidation group, and day 4 for the consolidation group. Thus, in theory it could be possible that no relationship was seen between the initial schema strength and old schema mean responses in the incongruent category for the consolidation group because the delay between IS and FT differed. However, this is unlikely since a) the measure of schema strength was recorded immediately after IS for both groups, b) the FT trials used for this analysis were learned 48 hours before the FT in both groups, and c) if any difference in the delay between IS and FT caused a significant correlation in the no-consolidation group but not the consolidation group for the inconsistent category, we would then also expect a lack of an effect in the correlation analysis for the consistent category for the consolidation group. Here, however, we found significant effects for both groups, consistent with our predictions. Moreover, we did not find any significant between group differences for items learned in the IS period with regards to performance in the FT. Specifically, for both the consistent and the inconsistent category, both groups had numerically comparable absolute errors (between group differences non-significant with *ps* > 0.498), and there were also no significant differences with regards to the ‘schema strength’ measure (difference between outside- and inside-quadrant trials) when assessed at the Final Test (between group differences non-significant with *ps* > 0.322), indicating that memory for IS items did not differ significantly between groups at the last day of testing.

Of further note, for the no-consolidation group the NL1 followed the IS phase on the same day, while the new material was presented on day 2 (in NL2) for the consolidation group. While the experimental set up (the same stimulus categories and the same background image) were identical between IS and new learning in both groups (NL1 for the no-consolidation group, NL2 for the consolidation group), it is still possible that the consolidation group was able to use any memory they had about the day at which an item was learned, to guide their response at the Final Test 48 hours later. Future studies could rule out this possibility by investigating whether comparing a short vs. long consolidation period (rather than a no consolidation vs. consolidation period as in the current study), confirms the observed between group differences. A replication of the current findings with such a design would also be advantageous, as such a design would reduce any potential effect of proactive interference during NL1 in the no-consolidation group.

Conceptually, the location schemas learned in the current study can be seen as a Bayesian ‘prior’ which restricts the location participants expect an item of a specific category to occur. Recent research in working memory has in fact demonstrated that participants incorporate such a prior of possible target locations when responding in a working-memory location task^26^. Category specific priors could serve as an important signal for mnemonic predictions. Our study is based on the implicit assumption that the mechanism underlying schema updating is a prediction error (PE) signal when first encountering the inconsistent material. PE-based learning is heavily studied in investigations of ‘belief updating’ in the context of reinforcement learning and decision making research, but less evidence exists for PE-based learning in memory tasks (though some progress has been made in this field^14,15,31,32^). Recently, it has been shown that reward prediction errors (RPEs) are positively related to later memory in an incidental episodic encoding paradigm^33,34^. Similar mechanisms may underlie learning based on prediction violations in schema memory and episodic memory-reward contexts: Research exploring the neurotransmitter systems involved in decision making suggest that in addition to reward-based memory enhancements, memory enhancements due to novelty are also related to dopaminergic processes^35,36^, suggesting that dopamine may also play a central role in schema updating due to inconsistent information.

Another reason to believe that overlapping mechanisms may indeed be involved in decision making/reinforcement learning and schema memory stems from reports of activation in similar brain areas, such as medial PFC, during performance of both kinds of tasks^19,37^. With regards to schema-memory, van Kesteren *et al*.^1^ proposed in their SLIMM (schema-linked inter- actions between medial prefrontal and medial temporal regions) framework that non-schema-related memories are encoded via the hippocampus and parietal connections, while schematized memories are encoded in a network centred around connections between MTL and MPFC. According to recent findings, schema memories depend mostly on frontal regions once they have been progressively schematized^38^. In contrast, more precise episodic memories are represented in posterior networks with a central role of parietal areas like angular gyrus (AnG)^23,39^. Whether AnG involvement decreases with greater schematization could provide new insight into the reorganisation of schematic memories on a neural level: Schematized memories might become less dependent on the posterior retrieval network with increasing schematization. Alternatively, posterior areas could start to represent items in a less detailed, more abstracted fashion. Some evidence for this latter suggestion comes from a recent study by Lee, Samide, Richter, and Kuhl^40^. The authors demonstrated that both specific item-level information as well as category level information could be decoded from AnG. Interestingly, specific item-level reactivation of encoded stimuli predicted correct rejections of similar, but unstudied items, whereas higher category-level reactivation (maybe more similar to ‘schematic’ memories) tended to predict false alarms to such items. This finding is consistent with evidence that AnG binds components of consolidated schemas into a coherent memory representation^41^. A demonstration that the same memories might rely on different structures, or that they are represented differently in the *same* structures with consolidation could provide powerful insights into how single episodes turn into schemas.

In sum, our findings are consistent with theories of knowledge acquisition and learning, which suggest existing consolidated schemas serve as a framework during new learning, facilitate the learning of consistent information, and lead to the modification and updating of schemas in case of erroneous predictions from previously learned, now out-dated schemas. We provide a novel paradigm that allows studying the dynamic establishment of schemas as well as schema updating over time. Variations of this paradigm (e.g., comparing a short vs. a long consolidation period or using electroencephalography to ‘directly’ observe prediction error) could further substantiate the current interpretation that enhanced PE-triggered schema updating is directly related to consolidation processes.

## Materials and Methods

### Participants

The study employed a between-group design with two groups (a non-consolidation group, and a consolidation group, see details below). The goal was to obtain data from overall 64 participants, with 32 participants per group. Prior to data analysis, data were screened for overall performance. Three participants (2 from the non-consolidation group, 1 from the consolidation group) were excluded because overall performance (as measured in absolute error across all trials in the Final Test, see below) was more than 2 *SD* below the mean performance of the entire group of 64 participants. These three participants were replaced with new participants prior to data analysis. The final sample thus consisted of 64 participants (44 female; 32 participants in the non-consolidation group, 32 participants in the consolidation group). Participants were 18–35 years of age (mean age = 24.1, *SD* = 3.79), fluent English speakers, had normal or corrected-to-normal vision, and had no history of psychiatric or neurological disorders. All methods were performed in accordance with the relevant guidelines and regulations of the University of Cambridge. Informed consent was obtained according to procedures approved by the Cambridge Psychology Research Ethics Committee and participants were paid £7.50/hour for their time.

### Materials

**Figure 5:**
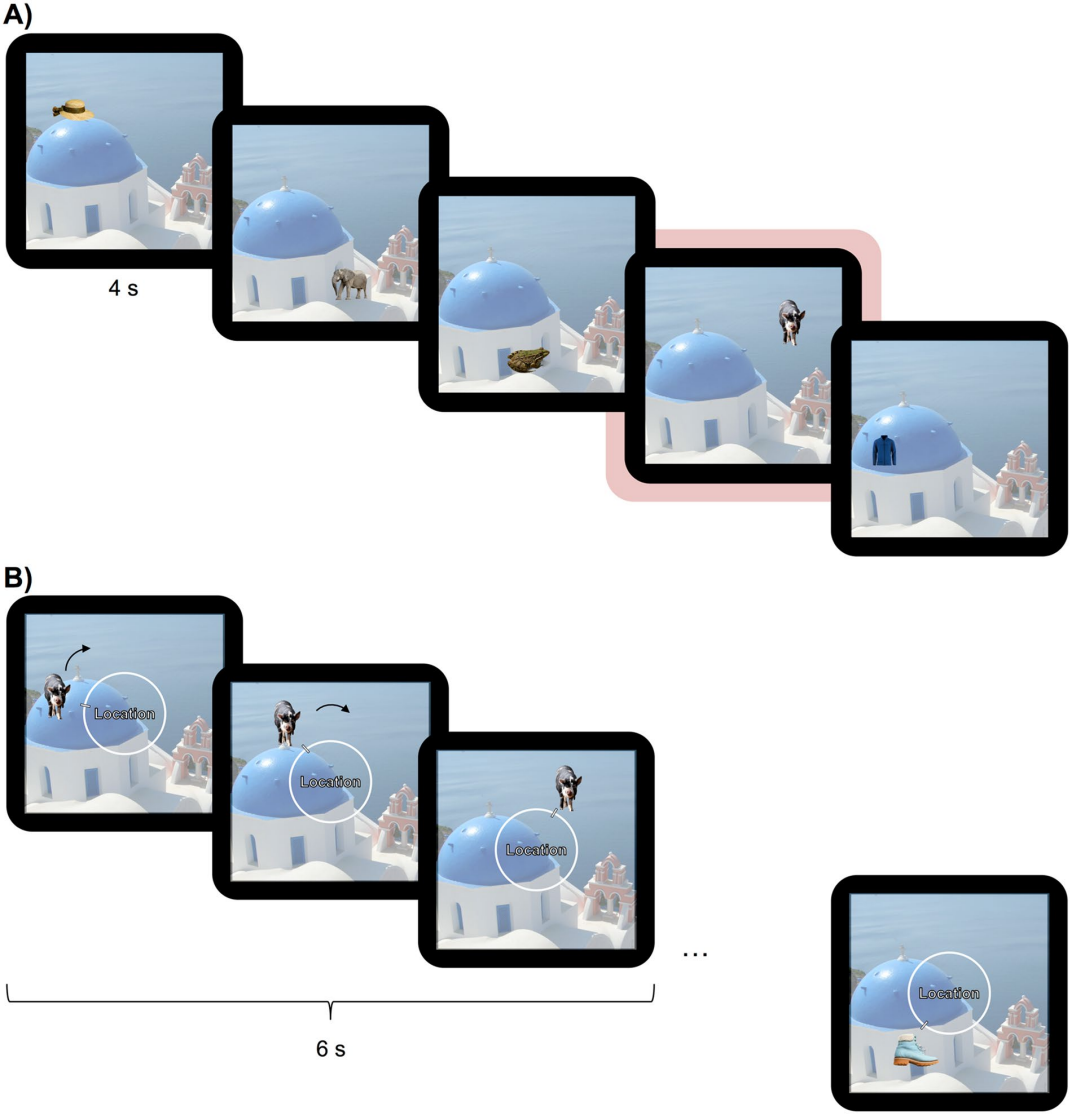
(**A**) Example stimuli presented in the study phases of the experiment (here for animals and clothing). Stimuli were presented for 4 s, followed by 500 ms of a fixation cross (not shown). While most stimuli of a category fit a schematic location (inside-quadrant trials), some pictures are presented outside of this category-specific circle segment (outside-quadrant trials, here underlaid in red). (**B**) Illustration of the continuous response task. The stimulus appeared at a random location on the screen and the participant had 6 s to move the stimulus on the circle (here indicated by arrows which were not present in the actual experiment) to the remembered location using the left and right arrow key. Each trial was separated by a fixation cross presented for 500 ms (not shown). In the FT phase objects from each of the four categories were tested intermixed. In the test phases after the study phases in each block of IS and NL1/NL2 only the stimuli (and thus two categories) learned in the immediately preceding block were tested. Image of Oia Church is a cropped version of the image from wikipedia.org, licensed under the Creative Commons Attribution-ShareAlike 3.0 License (https://creativecommons.org/licenses/by-sa/3.0/). All other images (hat, boot, frog, jacket, pig, elephant, backgrounds removed) were retrieved from pixabay.com, licensed under a Creative Commons CC0 License (https://creativecommons.org/publicdomain/zero/1.0/deed.en).

Stimuli consisted of 448 pictures from 4 different categories: animals, clothes, furniture, and food (fruits and vegetables). The stimuli were obtained via an internet image search. The background was removed for each picture and stimuli were presented in a size of ~150 × 150 pixels on top of a 750 × 750 pixel background image that was used on every trial throughout the experiment (see Fig. 5A). The location of the pictures on this background was restricted to any location on an invisible circle, similar to previous paradigms that studied memory precision for location information^20–23,42^. Circle locations were further restricted within the categories. For each category most of the stimuli (22 out of the 28 items per block; ‘inside-quadrant trials’) were presented within a 90-degree segment of the circle. These category specific 90-degree circle segments constituted the location schemas for the categories. The remaining subset of stimuli (6 out of the 28 items per block; ‘outside-quadrant trials’) was presented outside this segment with a minimum of a 30-degree distance to the inside-quadrant locations to create variance in the schemas. We aimed for roughly 20% outside-quadrant trials to ensure that the schema was not immediately recognized, but at the same time have enough inside-quadrant trials for stable schema development.

### Procedure

Upon arrival, participants completed a short training session to familiarize themselves with task requirements, including the continuous response options. The practice session consisted of 34 trials spread over 4 blocks of length 2, 4, 10 and 18 trials. Stimuli used in this training session were of a fifth category (musical instruments) and did not overlap with stimuli used in the main experiment. The study was designed as a between subject experiment. Regardless of the group participants were assigned to (based on which sessions they signed up to), all participants completed 4 phases across 3 experimental sessions. These phases comprised Initial Study (IS), New Learning on day 1 (NL1), New Learning on day 2 (NL2), and Final Test (FT).

Each participant learned all 112 stimuli of two categories, and only half of the stimuli (56) of the other two categories. The learning of the 112 stimuli was divided across phases. Specifically, each participant learned 56 images of these categories during the IS phase. The remaining 56 stimuli of these two categories were learned during NL1 or NL2, depending on group assignment. Thus, for these two categories (the categories that were already studied in IS; henceforth ‘relevant’ categories) all 112 images of the category were learned across the experiment. The remaining two categories (henceforth ‘irrelevant’ categories) constituted filler items, and only half the stimuli from these categories were employed in the experiment. These ‘irrelevant categories’ were included simply to keep the amount of information learned across the NL1 and NL2 phases consistent between the two experimental groups, but they were not relevant for our hypotheses. Thus, each participant learned a total of 336 stimuli out of the complete stimulus set of 448 stimuli.

Between groups we manipulated whether new learning of relevant information after the IS phase would occur without any possibility for consolidation (no-consolidation group) or after a delay allowing for consolidation, including a night of sleep (consolidation group). The critical comparisons included performance during the final memory test (last day) for objects from the above mentioned ‘relevant’ categories, which each participant learned during the New learning phase 48 hours before the Final Test (i.e., NL1 or NL2, depending on the group). In order to keep the temporal distance between the learning of this ‘critical’ information and the Final Test consistent at 48 hours (as mentioned above), we tested participants on the following schedule: Participants in the no-consolidation group came to the lab at the same time of day on three consecutive days, while participants in the consolidation group came to the lab at the same time on days 1, 2 and 4. On day 1, all participants completed the ‘Initial Study’ phase and a first ‘New learning phase’. On day 2 they completed a second phase of ‘New learning’. On day 3 participants in the no-consolidation group completed the ‘Final Test’, which took place on day 4 for participants in the consolidation group.

During IS each participant learned stimuli from one of the natural categories (animals or food) and one of the human-made categories (clothes or furniture). The categories learned during this and subsequent phases were counterbalanced across participants. In phases IS, NL1 and NL2, stimuli from both categories were intermixed across 4 blocks and a continuous response test followed each of the learning phases within the blocks. Figure 5A shows an example of the stimulus displays. Following each block, participants’ memory for the stimuli learned in the immediately preceding block was tested (see Fig. 5B for an illustration of the continuous response test used).

Each study and test phase in IS (as well as in NL1 and NL2, see below) consisted of 28 trials. In each study trial, participants were presented with the stimulus superimposed on the background for 4 s, followed by 500 ms of a fixation cross. Participants were instructed to try to learn the location of the objects on the display as precisely as they could. In each test trial, participants were tested on their memory for the location of a previously learned picture. The order of the pictures was randomized. Each tested object was initially presented in a random location on the invisible circle. The participants’ task was to recreate the pictures’ previous location using a continuous dial (360 increments). The word ‘Location’ was presented in the centre of the screen in white font. Participants had 6 s to recreate the location using the left and right arrow keys on the computer keyboard. They locked their answer using the space bar. The colour of the cue word ‘Location’ changed from white to red if no response was made within the first 4 s of the 6 s response window to encourage participants to respond.

**Figure 6:**
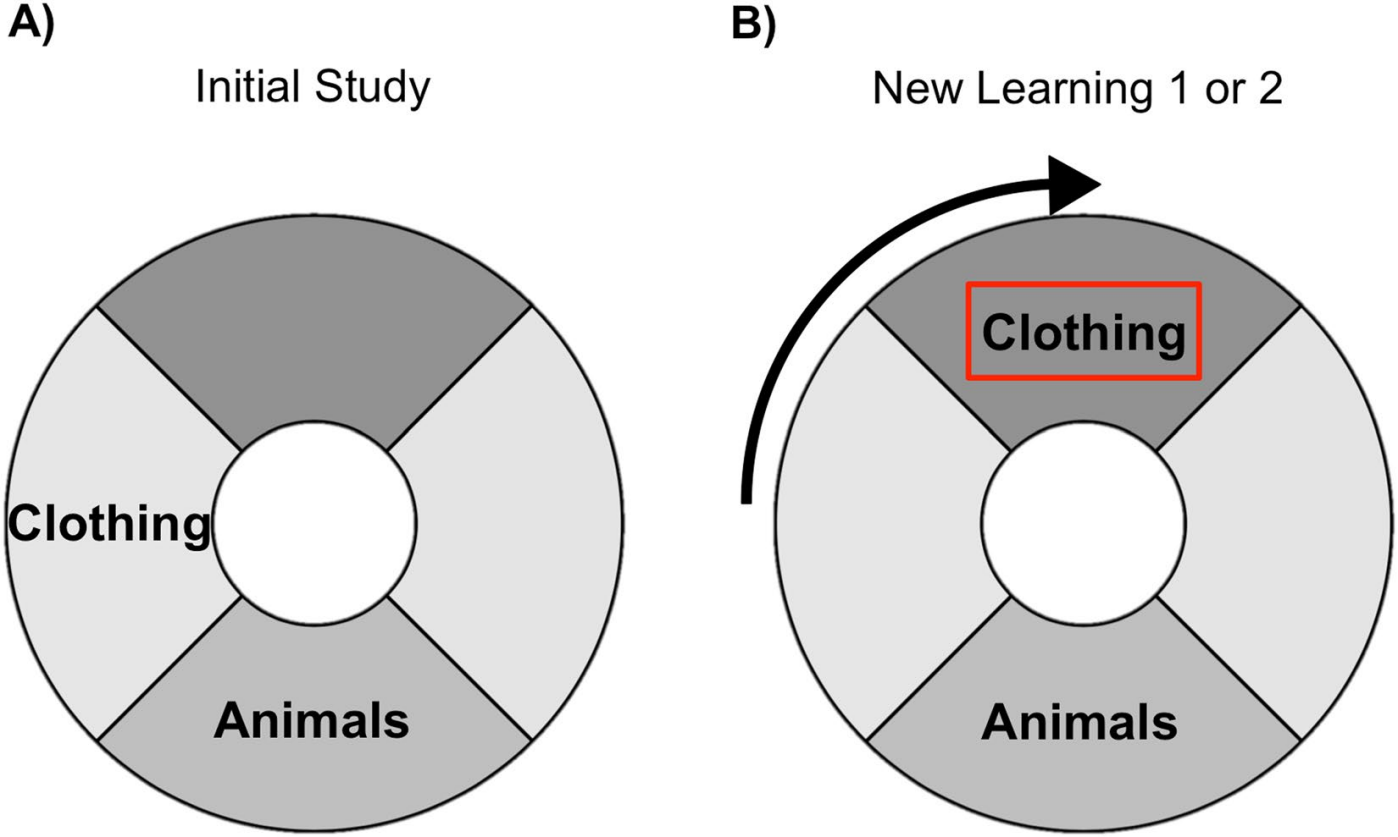
Illustration of the schema shift in the inconsistent category. Shaded quadrants illustrate category-schema specific areas of the circle. (**A**) In this example the inside-quadrant trials of the ‘clothing’ category were presented in the left quadrant of the circle during the IS phase. (**B**) During the New learning phase (NL1 for the no-consolidation group, NL2 for the consolidation group) the category shifted by 90 degrees clockwise so that now inside-quadrant trials in the ‘clothing’ category would be presented in the top quadrant of the circle.

The NL1 phase followed immediately after IS. In the no-consolidation group, participants were presented with new stimuli of the same categories as those learned during IS (‘relevant categories’). That is, if a participant had studied clothing and animals during the IS phase, they would now continue to study new clothing and animal stimuli. For one of these categories, the same location schema as during IS was used (‘consistent category’). For the other category, however, the location schema changed by 90 degrees clockwise (‘inconsistent category’). That is, if clothing was (mostly) presented at the left of the circle during IS, it would now be presented (mostly) in the top quadrant of the circle (see Fig. 6). (Note that, as described at the beginning of the results section, for all categories in the experiment, including the consistent categories there were both inside and outside-quadrant *trials*. That is, *category* consistency refers to the overall consistency of stimuli in this category across the experiment, but not to the trial level).

In contrast, the consolidation group was presented with stimuli from the *other* two (new/irrelevant) categories in the New learning phase immediately following IS on day 1. That is, if a consolidation group participant had studied clothing and animals during the IS phase, they would be presented with furniture and food (the two ‘irrelevant’ categories) in the NL1 phase.

During NL2 on day 2, in contrast, participants in the no-consolidation group learned stimuli from the two new, ‘irrelevant’ categories, while participants in the consolidation group learned new stimuli of the same, ‘relevant’ categories that they had encountered during IS, with one category being consistent, and the other one becoming inconsistent, as described above. Thus, the critical difference between the no-consolidation group and the consolidation group was which information was learned in the NL1 and NL2 phases. For the no-consolidation group, stimuli in the NL1 phase were of the same (‘relevant’) categories as the stimuli learned during IS, with one category being consistent with its previously learned schema and the other one being inconsistent, and the two new/irrelevant categories were learned in NL2. For the consolidation group, this order was reversed with irrelevant information learned during NL1, and relevant information learned during NL2.

The FT always occurred 2 days after the ‘new learning’ of the relevant stimuli, and therefore on day 3 for the no-consolidation group (who learned the relevant stimuli on day 1) and on day 4 for the consolidation group (who learned the relevant stimuli on day 2). In the FT participants did not encode any new stimuli, but were tested on the entire 336 stimuli that they had learned on the previous days. (Thus, while each stimulus was only studied once, it was tested a second time at Final Test). The testing procedure is illustrated in Fig. 5B. The 336 trials of the Final Test were distributed across 6 blocks. Order of the stimuli across these blocks was randomized. Stimulus presentation in all phases was done using Matlab (Mathworks, Natick, MA) and the Psychtoolbox (www.psychtoolbox.org).

### Data analysis

Data analysis was conducted using Matlab and SPSS (IBM Corporation). Data visualisation was done using Python using Jupyter notebooks in conjunction with a number of libraries including numpy, scipy, matplotlib, seaborn, and pandas.

#### Model Specification

All models are based on the ‘standard mixture model’, which has successfully been used to capture responding in long-term memory^21,23^. This model combines a uniform distribution capturing guessing, as well as a von Mises (circular Gaussian) distribution capturing target responses. Consequently, all models include as a minimum 2 free parameters: the probability of random response (probability of guesses) and the concentration parameter ‘Kappa’ of the von Mises distribution.

Model 1 (‘standard mixture model’): Model 1 is a standard mixture model, with the free parameters described above, applied to the Final Test data (critical items) from the consistent category.

Model 2 (standard + schema mean model’): Model 2 is an extension of model 1, which also models responses clustered around the ‘schema mean’, and thus includes a third free parameter: the probability of schema mean responses.

Model 3 (‘standard mixture model’): Model 3 is equivalent to Model 1, but applied to the Final Test data (critical items) from the inconsistent category.

Model 4 (‘standard + original/old schema mean model’): Model 4 is comparable to Model 2, but instead of modelling schema mean responses here responses to the ‘original’ (old/no longer relevant) schema mean is modelled in the Final Test data (critical items) from the inconsistent category. The third free parameter is thus the probability of old schema mean responses.

Model 5 (‘standard + new schema mean model’): Model 5 is comparable to model 4, but instead of modelling ‘old’ schema mean responses, ‘new’ schema mean responses are modelled (i.e., the schema mean that was relevant at the time of learning). The third free parameter is thus the probability of new schema mean responses.

#### Permutation Test

In order to statistically compare performance between the two groups in the inconsistent category we used permutation testing. Participants were randomly assigned to each of the two groups and the mixture model was fitted to each randomized groups’ pooled data over a total of 5000 iterations. For each of these iterations we calculated the between group difference. The resulting null distribution (as group assignment was random) was used to calculate the two-tailed *p*-value reflecting the proportion of iterations where the absolute group difference for the randomized groups’ data exceeded the observed difference between the no-consolidation and consolidation group.

#### Generalized linear model

Where the assumption of Sphericity was violated in reported ANOVAs, *p*-values based on adjusted degrees of freedom according to Greenhouse-Geisser will be reported with original *F*-values and original degrees of freedom, alongside the Greenhouse-Geisser Epsilon value.

## Data Availability

The code and data for all analyses will be made available in due course at https://github.com/BaMLaboratory.
